# Pulmonary Embolism: Hidden in the Disguise of Atrial Flutter/Atrial Tachycardia

**DOI:** 10.7759/cureus.70183

**Published:** 2024-09-25

**Authors:** Utsow Saha, Soumyadipto B Arko, Saiyara Sheikh Shama, Carlos Gonzalez

**Affiliations:** 1 Internal Medicine, Icahn School of Medicine at Mount Sinai, Queens Hospital Center, New York, USA; 2 Internal Medicine, Dhaka Medical College Hospital, Dhaka, BGD; 3 Internal Medicine, Anwer Khan Modern Medical College and Hospital, Dhaka, BGD

**Keywords:** arrythmia, atrial flutter, atypical ekg, case report, deep vein thrombosis (dvt), diagnostic challenge, hidden embolus, pe, pulmonary embolism (pe), therapeutic anticoagulation

## Abstract

Every year, pulmonary embolism (PE) causes about 100,000 fatalities in the United States. Acute PE is a prevalent and occasionally fatal kind of venous thromboembolism (VTE). PE can appear in a variety of ways and is frequently nonspecific in its clinical presentation, making its diagnosis challenging. In order to limit the associated morbidity and mortality, individuals with suspected PE should be evaluated efficiently. This will allow for a prompt diagnosis and administration of medication. An efficient and convenient method for diagnosing PE is to use an electrocardiogram (EKG). Remembering that the typical EKG for PE cannot always be present is essential. Sinus tachycardia, a fairly nonspecific EKG presentation, is the most common. In this case report, we present a 60-year-old male who exhibited signs and symptoms of chest pain and dyspnea, with EKG showing atrial flutter, and was ultimately diagnosed with PE as the underlying trigger. The objective of this case study presentation was to highlight the need to rule out PE in patients exhibiting dyspnea and chest pain, even in the absence of a traditional textbook EKG appearance. It is very crucial to consider the holistic presentation of the patient. Acute onset dyspnea and chest pain should always prompt PE as an important differential.

## Introduction

Pulmonary embolism (PE) presents a substantial challenge for patients with complex medical histories since diagnosis might be elusive [1-6]. Atypical presentations, such as mimicking arrhythmias like atrial flutter and tachycardia, further complicate diagnosis [6-11]. Even though a thorough examination and supportive investigation findings are the cornerstone for diagnosing PE, not every patient presents with the common symptoms, which often embarks challenges in the diagnosis and proper treatment of the disease, making it one of the biggest culprits of cardiovascular mortality. It is quite rare to come across a case of PE accompanied by atrial flutter, and even when encountered, it is often overlooked [11-14]. We hereby describe a case of a 60-year-old male with multiple comorbidities, including asthma, heart failure with reduced ejection fraction, and chronic obstructive pulmonary disease (COPD)/interstitial lung disease (ILD), who presented with acute dyspnea and suggestive electrocardiographic findings (right ventricle (RV) enlargement and RV free wall hypokinesis). Recognizing PE in patients with acute dyspnea and new-onset arrhythmias, especially those with complex medical histories, is crucial for optimal patient outcomes. Early identification and management are of paramount importance [1,6,9]. The present study presents an evaluation of the pathophysiology, clinical symptoms, diagnosis, treatment, prognosis, and follow-up of PE in the light of our case.

## Case presentation

**Figure 1:**
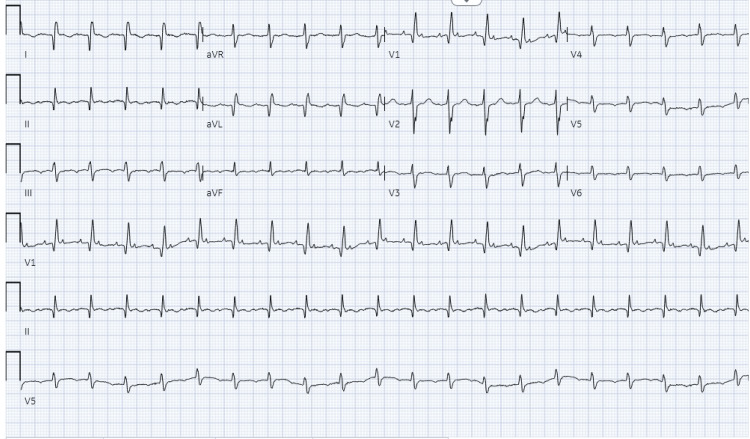
EKG-1 with atrial flutter/atrial tachycardia

**Figure 2:**
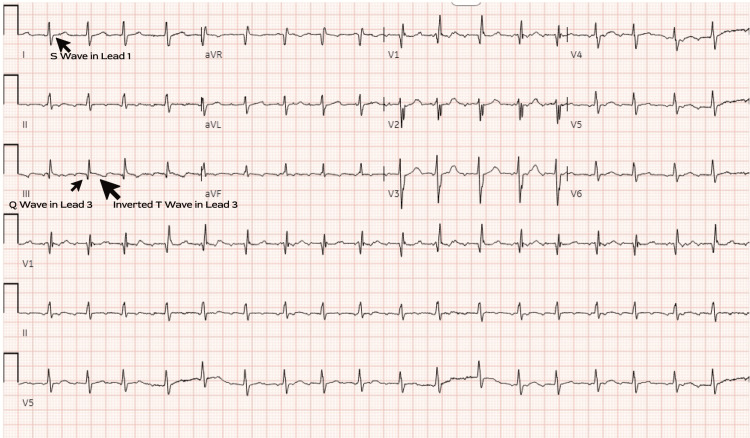
EKG-2 after slowing the heart rate and with S1Q3T3 pattern

A 60-year-old male with a history of allergic rhinitis and severe pulmonary hypertension (group 3), compounded by asthma, COPD, and ILD, presented to the emergency department with chest pain and worsening dyspnea on exertion. The patient reported chronic exertional dyspnea and generalized weakness over the preceding months, exacerbated on the day of admission. Notably, the patient reported remote bilateral lower leg swelling, which has since resolved. Hemoptysis was noted as a new symptom. Despite adherence to prescribed medications (short-acting beta agonist, inhaled corticosteroid, oral antihistamine), including home oxygen therapy, the patient was presented with tachycardia and tachypnea. His heart rate was 110 bpm, and his blood pressure was 100/70 mmHg on admission. Laboratory investigations revealed elevated pro-brain natriuretic peptide (BNP) and lactate levels. Electrocardiography demonstrated atrial flutter vs. atrial tachycardia (Figure 1), with subsequent troponin elevation and characteristic S1Q3T3 pattern on repeat electrocardiogram (EKG) (Figure 2).

**Figure 3:**
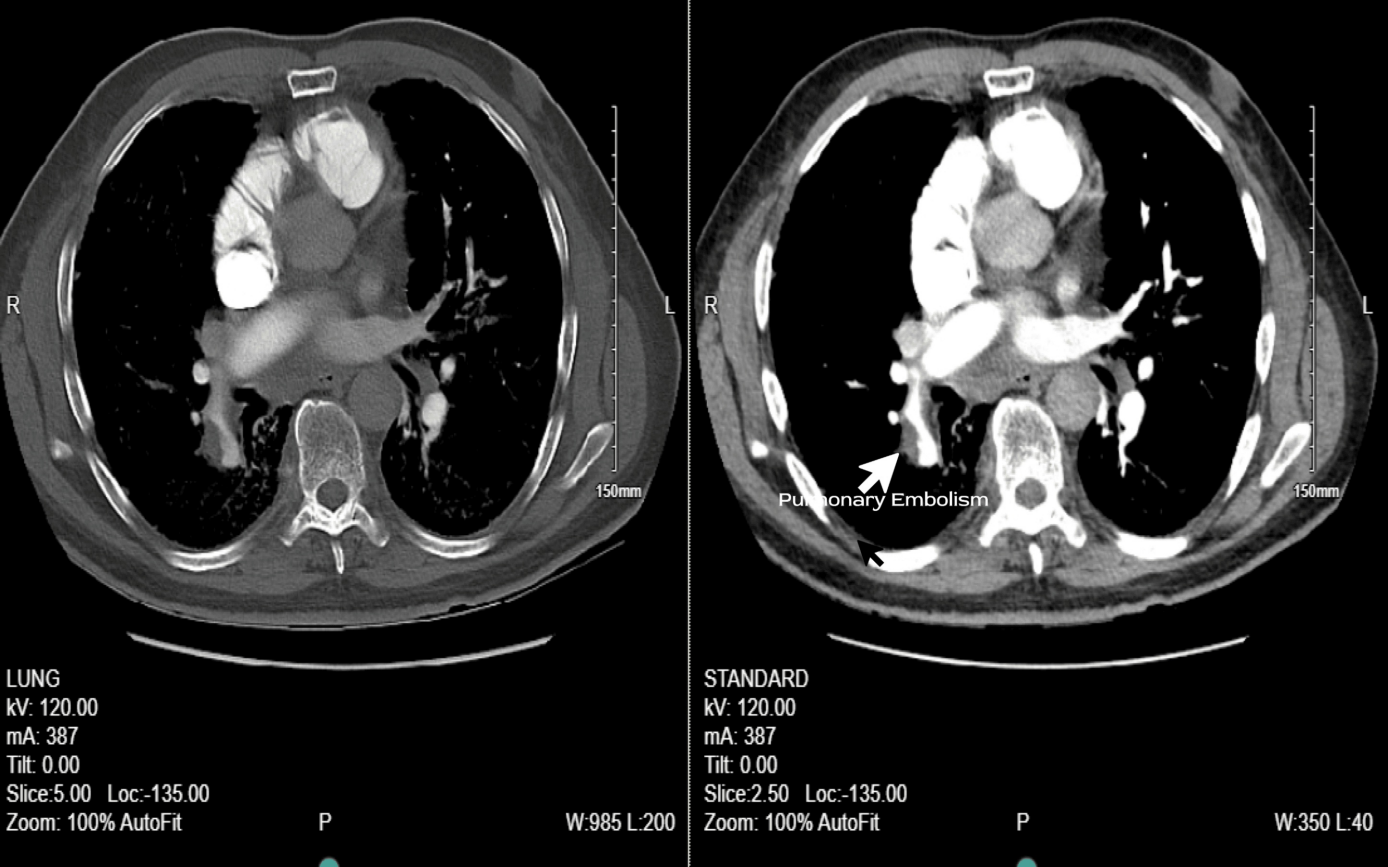
CT angiogram of pulmonary embolism with contrast Acute-on-chronic pulmonary embolism involving the right lower lobe pulmonary arterial segmental branches, with evidence of right heart strain and pulmonary arterial hypertension.

Given the high clinical suspicion for PE, prompt imaging with a chest CT angiogram (CTA) was performed, confirming the diagnosis (Figure 3).

The patient's Well score was three, which made him fall into the intermediate category. Therapeutic anticoagulation was initiated promptly. The patient responded well to this and recovered from this acute episode within three days. No thrombolysis was needed as the patient was hemodynamically stable.

## Discussion

The case of our 60-year-old male patient underscores the complexity of diagnosing PE in the setting of atypical presentations, particularly in individuals with multiple comorbidities. PE, although typically associated with dyspnea, tachycardia, and hypoxemia, can occasionally manifest as arrhythmias, such as atrial flutter and atrial tachycardia, leading to diagnostic challenges.

Definition

The term “pulmonary embolus” implies a blockage of the pulmonary artery or any of its branches caused by a clot that formed in a distant site of the body. 

Epidemiology

Since the advent of D-dimer testing and computed tomographic pulmonary angiography (CTPA) in the 1990s, estimates of the general population's PE incidence have risen. Trends related to overdiagnosis, such as a spike in incidence, no change in mortality, and a decline in case fatality, were linked to the implementation of the CTPA and D-dimer test [1]. We can now diagnose more pulmonary emboli - thanks to technological advancements. However, the incidence of PE has not altered much over the last few decades. 

Regarding the sex distribution of PE, the incidence rate is a little bit higher among males than among females (56 against 48 per 100,000 respectively) [2,3]. Among women in particular, the incidence increases with age; beyond 75 years of age, the incidence climbs to >500 per 100,000 [3]. In certain special populations, there is a substantially higher incidence of deep vein thrombosis (DVT) and PE [2]. When it comes to cancer patients, an estimated 10% of them usually develop clinically evident venous thromboembolism syndrome. The production of procoagulant factors by cancerous tissue and the use of chemotherapeutic agents both contribute to this phenomenon. The risk is also significantly amplified in acutely ill hospitalized patients with medical illnesses like cancer, stroke, etc. It is pertinent to be mindful that surgical patients admitted to the hospital encounter a greater likelihood of developing DVT and PE. Nevertheless, pregnancy and the postpartum period augment the risk of lower-limb DVT and PE, which might prove fatal. Other risk factors include acute traumatic spinal cord injury, nephrotic syndrome, total joint arthroplasty or replacement, and inherited thrombotic disorders.

The USA sees a staggering number of nearly 100,000 deaths each year due to PE, whereas, in Europe, PE kills roughly 300,000 people each year [2,4,5].

Pathogenesis and pathophysiology

The pathogenesis of PE is linked to the generation of a thrombus (also known as Virchow’s triad) [6]. Virchow's triad comprises venous stasis, endothelial damage, and hypercoagulable condition. The majority of emboli are believed to originate from the proximal veins of the lower extremities, namely, the iliac, femoral, and popliteal veins, and more than half of patients with proximal vein DVT had concomitant PE upon presentation [6].

Pulmonary infarction occurs when tiny thrombi occlude distally into the segmental and subsegmental arteries, affecting around 10% of patients. This manifests as pleuritic chest pain and hemoptysis, which is thought to be caused by a severe inflammatory reaction in the lung and the surrounding visceral and parietal pleura [6]. When hemoptysis comes along with pleuritic pain and hypoxemia, it is important to consider the possibility of acute PE. However, it is worth noting that it can also be caused by pneumonia or heart failure. Thus, it is always good to keep an open mind and consider all the possibilities. It is very likely to have PE if one has sudden chest pain, especially pleuritic pain, although it can be caused by pneumonia, pleuritis, or pericarditis. Another consequence of PE is impaired gas exchange, which is caused by obstruction of the pulmonary vasculature. This ultimately results in the alteration of the ventilation-to-perfusion ratio. Furthermore, functional intrapulmonary shunting is triggered by surfactant dysfunction and atelectasis as a result of inflammation. The aforementioned complications eventually culminate in hypoxemia [6]. Hypocapnia and respiratory alkalosis are the ultimate consequences of increased respiratory drive caused by PE. If PE leads to circulatory shock, even hypercapnia and acidosis may ensue.

**Figure 4:**
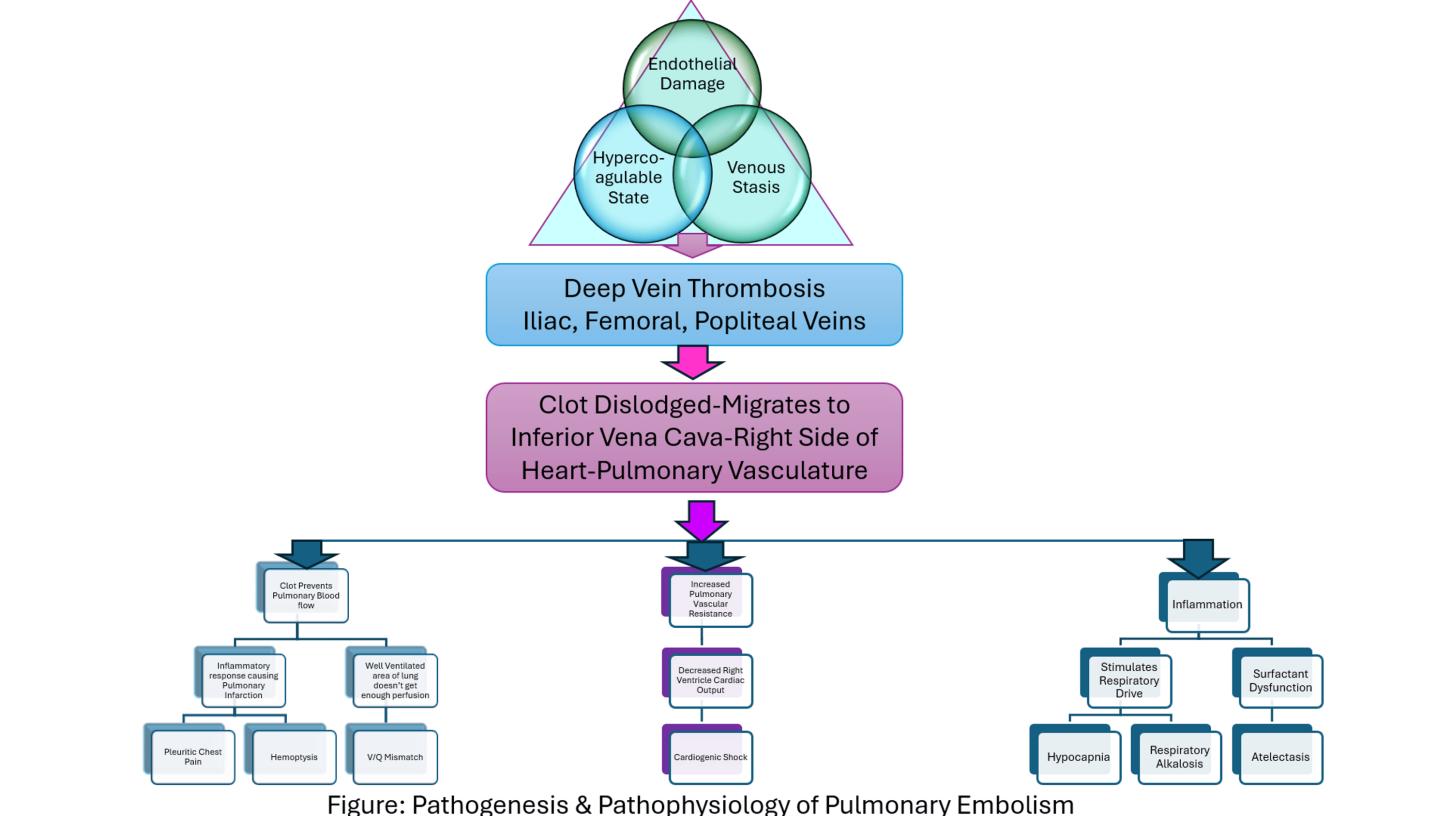
Pathogenesis and pathophysiology of pulmonary embolism This illustration was made by the authors based on the source [6].

Physical blockage of the vascular bed by thrombus raises pulmonary vascular resistance (PVR). Increased PVR interferes with right ventricular outflow, resulting in a dilated RV and bowing of the intraventricular septum. Both reduced blood flow from the RV and RV dilatation decrease left ventricular preload, diminishing stroke volume and, consequently, cardiac output [6]. The pathogenesis and pathophysiology of PE are illustrated in Figure 4.

The diagnosis of PE is seldom challenging because of its extensive spectrum of clinical presentation. There is a vast spectrum of symptoms that PE can present with, from complete absence to shock and even sudden death. PIOPED (Prospective Investigation of Pulmonary Embolism Diagnosis) II demonstrated the most prevalent symptoms among patients with PE [6] - dyspnea at rest or with exertion (79%), pleuritic pain (47%), cough (43%), calf or thigh pain (42%), calf or thigh swelling (39%), orthopnea (36%), wheezing (31%), chest pain (not pleuritic) (17%), calf and thigh pain (16%), calf and thigh swelling (8%). Our patient presented with worsening dyspnea with exertion, along with chest pain and hemoptysis. His previous history was significant, with bilateral leg swelling, which had resolved. 

Hemodynamic collapse, presyncope, syncope, and transient or persistent arrhythmias (such as atrial fibrillation), hoarseness due to Ortner syndrome are less frequent presentations [7]. Dyspnea is a frequently (albeit not always) evolving condition, typically occurring within seconds (46%) or minutes (26%) [6].

According to the National Collaborative Study PIOPED II, the most common presenting signs on examination are the following [6]: tachypnoea (20/minute) (57%), either calf or thigh edema, tenderness, erythema, palpable cords (47%), tachycardia (/minute) (26%), crackles (rales) (21%), diminished breath sounds (21%), accentuated P2 (pulmonary component) of the second heart sound (15%), distended jugular vein (13%), both calf and thigh edema, tenderness, erythema, palpable cords (12%), right ventricular lift (5%), diaphoresis (4%), rhonchi (5%), wheeze (3%), temperature (>101.3°F) (2%). After a thorough physical examination of our patient, tachycardia and tachypnea were found.

Surprisingly, numerous patients, including those with a significant PE, may not experience any symptoms at all or only exhibit nebulous or ambiguous ones, which makes this very tricky(8). Physicians frequently confront the dilemma of whether to pay attention to PE or skip expensive and potentially hazardous evaluation techniques like computed tomography. Maintaining a high degree of suspicion is, therefore, essential to ensuring that clinically significant instances are not overlooked.

Laboratory investigations and diagnosis

Routine laboratory tests may reveal leukocytosis, a high erythrocyte sedimentation rate (ESR), a rise in aspartate aminotransferase (AST), serum lactate, and lactate dehydrogenase (LDH). However, all of these are nonspecific findings. The serum level of BNP or its precursor, Pro-BNP, and troponin carry more prognostic value than diagnostic value [6,7].

When encountering unexplained hypoxemia over a normal chest radiograph, it is imperative to have an urgent clinical suspicion of PE and swiftly conduct further investigations. It is really crucial to go through the arterial blood gas (ABG) analysis in these patients as ABGs are abnormal in most of the patients assumed to have PE [8-10]. ABGs often showcase a variety of common abnormalities. These can include one or more of the following: hypoxemia, widened alveolar-arterial (A-a) gradient for oxygen, hypocapnia, and respiratory alkalosis [9]. It is quite rare, but in some cases, massive PE might be associated with obstructive shock and respiratory arrest. If so, patients may experience hypercapnia, lactic acidosis, respiratory acidosis, or both lactic and respiratory acidosis. However, it is worth noting that ABG results can still fall within the normal range in up to 18% of people with PE [9]. Unusual levels of oxygenation could potentially provide valuable insights for prognosis. For instance, if room air pulse oximetry readings are below 95% at the time of diagnosis, they are at higher risk of facing grave consequences. These complications can include obstructive shock, respiratory failure, and, unfortunately, even death [6].

It is essential to calculate the pre-test probability (PTP) for PE whenever there is a suspicion of PE [6,8]. Wells criteria are preferred to determine the probability into three segments - low (<2), intermediate (2-6), and high (>6). The Wells criteria encompass a number of elements: three points for clinical signs and symptoms suggestive of DVT or PE is the number one likely diagnosis, 1.5 points for tachycardia (heartbeat > 100/minute), previous objectively diagnosed history of DVT or PE, history of undergoing surgery within past four weeks, or immobilization for at least three days. Hemoptysis or malignancy (receiving treatment, treatment ended within six months, receiving palliative care) carry one point each. Our patient's Well score was three.

Although D-dimer assays are impressively sensitive, their specificity tends to be on the lower side, typically ranging between 40% and 60%. While an elevated D-dimer alone cannot unambiguously diagnose definite PE as this may be falsely elevated in acute illness, any recent trauma or surgery, or rheumatological disease, a normal D-dimer below 500 ng/mL (fibrinogen equivalent units) can be quite handy in ruling out PE for patients with a low or intermediate likelihood of having this condition, and no additional testing is usually recommended in these instances [10]. When the D-dimer level crosses the cutoff mark of 500 ng/mL (fibrinogen equivalent units) in intermediate-risk patients, it is time to whip out the diagnostic imaging, preferably with CTPA [6,8]. Catheter-based pulmonary angiography or magnetic resonance pulmonary angiography are not recommended first-line imaging modalities. Furthermore, while dealing with patients who are at a high risk of PE, a normal D-dimer test is not as useful for ruling out the diagnosis and, therefore, is not necessary. CTPA is recommended to be performed in patients with a high probability of PE or those with a low to moderate likelihood of PE, but laboratory investigation reveals an elevated D-dimer level [6,8,10].

CTPA is the preferred imaging modality when needed. PE is diagnosed by a filling defect that shows up after contrast enhancement in any branch of the pulmonary artery. The V/Q scan is a great alternative for patients who may not be able to undergo a CTPA due to particular circumstances, which include moderate or severe contrast allergy and a high probability of contrast nephropathy [6,8]. When CTPA is not conclusive or when further testing is required, for example, when the clinical suspicion of PE is still high even after negative imaging - a V/Q scan may also be recommended. Normal ventilation combined with either a segmental or subsegmental perfusion defect is indicative of PE [8,10]. In our case, we did not perform the D-dimer test; rather, we directly obtained the CTPA of the patient because of the high probability.

EKG abnormalities are extremely variable, even though they are frequently seen in patients with suspected PE [11]. Rhythm disturbance has been observed in up to 35% of patients. There are certain findings that are quite common in these scenarios, such as tachycardia and nonspecific ST-segment and T-wave abnormalities, which make up a whopping 70% of the most prevalent findings. It is quite puzzling that abnormalities that were once thought to indicate PE, such as the right ventricular strain, S1Q3T3 pattern, and new incomplete right bundle branch block, are, in reality, quite rare, occurring in less than 10% of instances [12]. Atrial fibrillation or atrial flutter, nevertheless, was absent in all patients without a history of cardiopulmonary disease. All patients with right bundle branch patterns or SlQ3T3 had their abnormalities resolved within 14 days [12]. Patients with PE who experience atrial tachyarrhythmias are more likely to endure a poor prognosis with a greater likelihood of mortality and morbidity [13]. Patients who experienced a fatal outcome were found to have significantly higher rates of atrial arrhythmias, primarily atrial fibrillation or flutter, complete right bundle branch block (RBBB), peripheral low voltage, pseudo infarction pattern (Q waves) in leads III and augmented vector foot (aVF), and ST segment changes - either elevation or depression over the left precordial leads [14]; 29% of patients exhibiting at least one of the aforementioned conditions on admission could not survive until hospital discharge [14].

It is still unclear whether right ventricular wall distension or myocardial ischemia is the root cause of this correlation despite the substantial connection. Multiple studies have provided empirical evidence that the use of an EKG for diagnosing PE is insufficiently trustworthy, given the extensively diverse manifestations of the condition. Thus, in conjunction with the clinical history and physical examination, ventilation-perfusion scintigraphy or pulmonary angiography must be performed to establish the diagnosis [15].

Management

There are three distinct categories in PE - massive, submassive, and low risk - which are distinguished either by the presence or absence of certain indicators, such as right ventricular dilation or dysfunction and hypotension [16]. The risk of mortality is directly linked to this categorization [17]. In the case of hemodynamically unstable patients, an important aspect to consider is the initial approach, which should prioritize the restoration of tissue perfusion through intravenous (IV) fluid resuscitation. If the patient does not improve even with IV fluid resuscitation, vasopressors need to be initiated as early as possible [16,17]. We must provide supplemental oxygen alongside. In addition to that, intubation and even mechanical ventilation may be required to stabilize the airway.

When a patient has a high suspicion of PE, rapid anticoagulation is preferred as long as there is no contraindication and definitive diagnostic imaging is performed, which is typically a CTPA [18,19]. Immediate anticoagulation is not preferred in patients with an intermittent and low-risk probability of PE [19]. When feasible, early ambulation should be recommended in most patients with acute PE as long as the patient is definitively treated. Bed rest may be required for the first 12 to 24 hours in patients with a high clot burden and severe PE to achieve optimum therapeutic anticoagulation. We treated our patients according to the current guidelines.

Prognosis

The prognosis of PE is variable. Nevertheless, total mortality from PE is generally higher if untreated (up to 30%) than if anticoagulation is used (between 2% and 11%) [20]. According to one study based on data from the WHO mortality database, the number of deaths decreased from 12.8% per 100,000 to 6.6% per 100,000 between 2000 and 2015 [5].

Monitoring and follow-up

Patients diagnosed with PE must be monitored for the following. Activated partial thromboplastin time, or aPTT, is the most widely used laboratory test for tracking unfractionated heparin. Its goal range is 1.5 to 2.5 times the upper limit of normal [18,19]. The prothrombin time (PT) ratio, which is typically stated as the international normalized ratio (INR) with a goal INR of two to three (target 2.5), is used to monitor warfarin [19]. The primary contributory factor of recurrent venous thromboembolism during therapy is inadequate anticoagulation. When required, the doctor should test for therapeutic anticoagulant levels while also taking into account other possible causes of recurrence.

## Conclusions

PE continues to be an enormous contributor to the overall disease burden worldwide. Our case highlights the importance of considering PE in the differential diagnosis of patients with acute dyspnea and new-onset arrhythmias, particularly in those with multiple comorbidities. Despite the challenges associated with atypical presentations, early recognition and management of PE are essential for optimizing patient outcomes. Following any of the clinical predictive rules is advocated for the accuracy of the diagnosis. Enhanced multidisciplinary teamwork is necessary to maximize the in-hospital management of patients with acute PE.
